# Future Trends for In Situ Monitoring of Polycyclic Aromatic Hydrocarbons in Water Sources: The Role of Immunosensing Techniques

**DOI:** 10.3390/bios9040142

**Published:** 2019-12-10

**Authors:** Shifa Felemban, Patricia Vazquez, Eric Moore

**Affiliations:** Sensing & Separation Group, School of Chemistry and life Science Interface, University College Cork, Tyndall National Institute, T12 R5CP Cork, Ireland; shifa.felemban@tyndall.ie (S.F.); patricia.vazquez@tyndall.ie (P.V.)

**Keywords:** polycyclic aromatic hydrocarbons (PAHs), nanomaterials, electrochemical immunosensor, environmental monitoring

## Abstract

Polycyclic aromatic hydrocarbons (PAHs) are hazardous environmental pollutants found in water, soil, and air. Exposure to this family of chemicals presents a danger to human health, and as a result, it is imperative to design methods that are able to detect PAHs in the environment, thus improving the quality of drinking water and agricultural soils. This review presents emerging immunoassay techniques used for in situ detection of PAH in water samples and how they compare to common-place techniques. It will discuss their advantages and disadvantages and why it is required to find new solutions to analyze water samples. These techniques are effective in reducing detection times and complexity of measurements. Immunoassay methods presented here are able to provide in situ analysis of PAH concentrations in a water sample, which can be a great complement to existing laboratory techniques due to their real-time screening and portability for immunoassay techniques. The discussion shows in detail the most relevant state-of-the-art surface functionalization techniques used in the field of immunosensors, with the aim to improve PAH detection capabilities. Specifically, three surface functionalization techniques are key approaches to improve the detection of PAHs, namely, substrate surface reaction, layer-by-layer technique, and redox-active probes. These techniques have shown promising improvements in the detection of PAHs in water samples, since they show a wider linear range and high level of sensitivity compared to traditional PAH detection techniques. This review explores the various methods used in the detection of PAH in water environments. It provides extra knowledge to scientists on the possible solutions that can be used to save time and resources. The combination of the solutions presented here shows great promise in the development of portable solutions that will be able to analyze a sample in a matter of minutes on the field.

## 1. Introduction

One of the common environmental pollutants in water and soil includes polycyclic aromatic hydrocarbons (PAHs) [1,2,3]. PAHs fall under a group of organic compounds comprised of hydrogen and carbon [4,5]. Common health and economic concerns that emerge from PAH pollutants include the potential of being teratogenic, carcinogenic, and mutagenic [6], all of which increase the risk of cancer among exposed populations. In humans, the uptake of PAHs can occur through various routes: Contact, ingestion, and inhalation [7,8,9]. Health impact of PAHs relies highly on the type of contact route, PAH concentration, and contact time [10]. The consequences of exposition to these pollutants are shown in Table 1.

Metabolic processes involving PAHs lead to the production of toxic metabolites that can attach to DNA, resulting in gene mutation [7]. Their potential impact, induced by cell damage and other biochemical disruptions, contribute to development of cancer, tumors, and other mutations [14]. The biological effects caused by PAHs on animals, mainly on aquatic organisms, have been of great concern to date. In particular, recent studies investigated the metabolic alterations (i.e., oxidative stress, hypoxic stress, neurotoxicity, changes in energy metabolism) induced by PAHs found in natural environments [17].

Benzo(a)pyrene (BaP) is one of the most significant PAHs reported to be highly carcinogenic, both in humans and animals. Different PAHs are grouped as causing cancer to animals, while some PAH-rich mixtures are grouped as cancer-causing substances to humans [18,19]. The Environmental Protection Agency identifies up to 16 PAHs as highly toxic and carcinogenic [20]. In the European Union (European Union under the Drinking Water Directive [21,22]), the limit of concentration values are set to 0.1 µg/L as the maximum total levels of PAHs allowed in water. In addition, there are specific limits for individual PAHs, as seen in Table 2.

**Figure 1:**
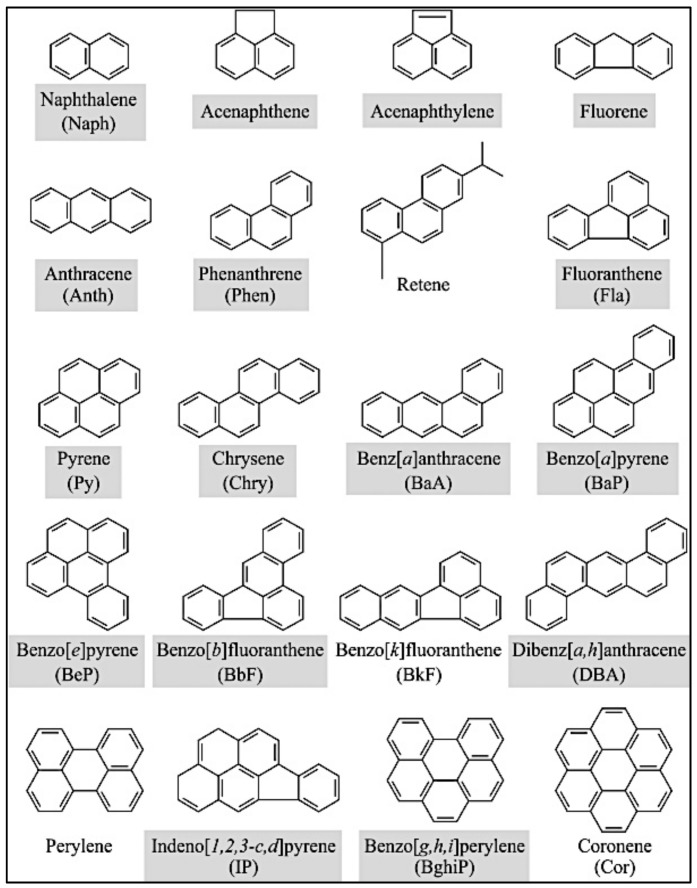
Common polycyclic aromatic hydrocarbons (PAHs) [23].

The most common PAHs found in the environment are illustrated in Figure 1 [23]. As shown, polycyclic aromatic hydrocarbon compounds present two or more fused aromatic rings within their chemical structure formation [5,6,24].

PAHs are highly nonpolar and lipophilic, and therefore can easily stick to plastics [25]. This fact forces researchers to use glasses for sampling and storage. For example, in collecting PAH samples for laboratory analysis, Webster et al. [26] noted that plastic materials should not be used for storage and sampling due to “the possible adsorption of the PAHs onto the plastic container material”. PAHs’ physical properties are important in regard to water pollution, as they do not mix well in water and manifest themselves as sediment. Nevertheless, as the number of fused rings decreases, vapor pressure reduces, resulting in increased adsorption of PAHs in water [25]. At room temperature, they present a solid appearance, as well as high boiling and melting points [27]. Examples of PAHs are: Naphthalene, with two rings; fluorine, with three rings. Four-ring PAHs include benzo(a)pyrene and chrysene, whereas benzo(ghi)perylene contains five rings.

Their presence in the environment is originated from mainly three sources. The first one, pyrolytic, occurs by the incomplete combustion of fossil fuels (petroleum and its derivatives, coal tar), waste incineration, and natural events such as forest fires and volcanic eruptions (although these do not have a significant contribution to the overall production of PAHs) [28]. The second group, petrogenic, derives from the presence in water of petroleum sources, such as crude oil and petrochemicals (gasoline, diesel fuel, kerosene, and lubricating oil). Finally, diagenetic processes contribute to the creation of PAHs by natural processes in the degradation of plants [29,30,31].

Research in the field of environmental monitoring has studied the footprint that these different sources present as contaminants. Each source (i.e., pyrolytic, petrogenic, and diagenetic) gives rise to typical PAH patterns. In general, combustion products are dominated by relatively high molecular weight (HMW) compounds with four or more condensed aromatic rings, whereas bi- and tricyclic aromatic compounds of low molecular weight (LMW) are more abundant in fossil fuels, which are, moreover, dominated by alkylated derivatives [32].

Analysis of water sources shows that it is possible to predict the cause of pollution: A phenanthrene-to-anthracene ratio (Phe/Ant) greater than >15 indicates that the source of PAHs is petrogenic (petroleum) in nature, while a ratio lowers than 10 indicates that PAHs are pyrolytic in origin [33]. Similarly, a ratio of 0.20 and less in BaA/(BaA + Chr) (benz(a))anthracene plus chrysene indicates that PAHs are petrogenic in origin, a range of 0.2–0.35 indicates a mixture of sources between pyrolytic and petrogenic, and a ratio of >0.35 shows pyrolytic origin [33]. Table 3 shows PAH studies in various regions of the world in relation to their proportion and original source.

More recent papers have successfully used different ratios to predict the sources of PAH pollution worldwide, both in surface water and sediment samples [17,42]. The dispersal of PAHs into the marine environment can occur through spills, surface runoff, industrial discharge, and wet and dry deposition [43]. In some cases, PAHs are adsorbed as particulate matter in water, and in the process can precipitate at the bottom of rivers and lakes [44]. Low molecular weight PAHs (2–3 rings) usually show a higher concentration in water than high molecular weight PAHs (4–6 rings). This is consistent with their properties. This is consistent with their properties as LMW PAHs have higher water solubility and vapor pressure than HMW PAHs [45].

## 2. Existing Challenges with Conventional PAH Analysis Methods

PAHs occur in different forms and concentrations in environmental areas such as water, air, and soil, which require the application of different methods of analysis depending on each particular case [19]. Presently, the common traditional analytical methods used to detect PAHs include, gas chromatography/mass spectrometry (GC/MS), including chemical ionization MS, ion trap MS, TOF/MS, and isotope-ratio MS (IRMS), and high-performance liquid chromatography (HPLC) with fluorescence detection or ultraviolet detection (HPLC/UV) [46]. In liquid chromatography (LC), practical limitation is in its ability to only detect a few dozen components, because of the limited peak capacity of its columns. HPLC is used for the detection of PAHs in water, but the decreasing popularity of LC can be seen in the withdrawal of method ASTM D4657-92 (standard test method for PAHs in water), a HPLC method for 16 PAHs in water. The simple use and compatibility of GC/MS are additional reasons for selection of gas chromatography in preference to LC for the detection of PAHs in water samples. The retention and separation of PAHs by GC method is affected by such conditions as solvent type, solvent effects, amount, injection conditions (speed, liner and sample size, temperature), and temperature programming. It should be pointed out that long retention times are normally required to obtain the resolution needed for the precise quantification of the HMW PAHs, although use of fast GC may lead to shorter analysis times.

There are several advantages that are associated with the use of these standard analysis techniques, such as accuracy, reliability, and sensitivity. They present, however, some limitations. Some of these disadvantages include the fact that their processes involve high-risk loss of analytes during sample separation, in addition to using an extensive amount of hazardous organic solvents [47]. This is a problem because it increases costs and contaminates the environment. Loss of analytes presents a major source of poor quality analytical data for PAH due to separation, pretreatment, and sampling of analytes, introducing errors that are not associated with the last quantification step. In addition, the application of these methods makes the procedure of sample analysis, preparation, and quantification quite time-consuming and tedious [48]. In addition to this, Lux et al. [49] pointed out that these methods require sophisticated equipment and present a lack of real-time detection.

When studying several groups of PAHs simultaneously, the extraction process can be complicated when PAHs of similar properties are included in the sample, as similar characteristics make the identification process difficult [50]. Molaei et al. [46] note that alternative approaches like single-drop microextraction, solid-phase microextraction, and stir bar sorptive extraction methods have been introduced to reduce costs, pollution, and shorten processing time. Manoli et al. [51] discussed a new model of magnetic solid phase extraction (MSPE). The MSPE process uses the magnetic material as adsorbents and comes with several advantages, like detecting PAHs in crude samples.

For decades, using a highly reliable GC/MS method solved the difficulty of detecting PAHs in crude samples. However, GC/MS has its limitations. Generally, even though GC/MS is extremely precise and sensitive, it is a very expensive equipment and therefore not available to all laboratories and consumers [52]. With the various limitations of the traditional chromatographic techniques, there is demand for low-cost, sensitive, rapid, on-site, environmentally as well as human friendly techniques that can be carried out by public healthcare providers and institutions who are keen to improve the quality of water [50]. Thus, this is where new emerging techniques like immunoassay come in.

## 3. Emerging Technologies

Besides conventional PAH detection methods, impact of pollutants on environmental water has also been monitored using biological systems like biomarkers and bio-monitors [53]. These biological techniques can measure the impact of anthropogenic activities to ensure compliance with environmental guidelines and regulations [54].

Biomonitoring involves the use of indicator organisms, for instance, filter-feeding mollusk bivalves [55,56]. As such, this technique is important because it can highlight the early presence of water pollutants entering water sources [17,57,58,59,60,61]. Bio-indicators are useful to explore the biological effects induced by PAHs, as well as the need to understand the mechanisms involved in the formation of this PAHs in our environment.

On the other hand, biomonitoring can only be used to detect the presence of pollutants, but not to identify or quantify a specific pollutant in water [62]. As a result, biomonitoring is limited in its application, and compared to other state-of-the-art methods such as immunoassay techniques, does not provide a persuasive performance [63]. In addition, another important disadvantage is their lack of reproducibility [64,65,66]. Given the limitations of these biological indicators in identifying PAHs among other pollutants, this review will not focus in their latest developments. In contrast, recent advances in immunoassay technology have offered promising results in this area. Immunoassays measure the presence of analytes in substances as a quick detection method. The major advantage of this technique is that it is reliable and highly selective, in addition to the possibility of miniaturization. Unlike other methods, integration with microfluidic systems makes it possible to conduct real-time field tests. For this reason, the following discussions will be based in state-of-the-art of immunoassay methods used in the detection of PAHs, as discussed below. The focus of this article is on the immunosensors of PAHs and on recent advances that use electrochemical sensing technology based on surface functionalization techniques. The advantages and disadvantages of surface functionalization techniques and their use for environmental monitoring will be presented here.

## 4. Immunoassays and Immunoassay Kits

Immunoassays use bioanalytical processes in the detection of analytes like lipids, carbohydrates, and proteins in solutions through the interaction of an antibody (an immune protein) [67]. Years of studies have largely advanced the development of immunoassays that are now used to detect PAHs in soil and water samples. Some available immunoassays can be used to determine PAH concentration; examples of this include chemiluminescence immunoassays (CLIA), fluoroimmunoassays (FIA), radioimmunoassays (RIA), and enzyme–linked immunosorbent assays (ELISA).

These examples differ mainly in the form of detection, be it UV, fluorimetric, amperometric, or potentiometric [68]. There is quite a variety of available commercial immunoassay kits used in PAH detection. Some of them target PAH detection by their physical characteristics (size, weight, etc.). For instance, BTEX RaPID Assay^®^ is a rapid field or laboratory enzyme immunoassay (from JJS technical services) that is able to analyze small aliphatic PAHs and carcinogenic PAHs (>4 aromatic rings) [69].

Commercial immunoassays can also be specific on the nature of the sample (soil, water). Ensys PAH is a soil test kit that can be purchased from Strategic Diagnostic Inc. The kit is used both as a qualitative and semi-quantitative assay, as it gives only an approximate value of the PAH concentrations present in soil [70]. Rapid Assay^®^ PAH test kit (from Tigret Inc., London, UK) is used for qualitative, semi-quantitative, and quantitative analysis of PAHs in water (well water, surface water, and groundwater) [71]. Envirogard^®^ (by JSS Modern Water, Schaumburg, IL, USA) also detects PAH in water, and it includes the 16 PAH compounds listed under the Environmental Protection Agency (EPA) regulations. These kits are both rapid and reliable [72]. Nevertheless, immunoassay techniques have their own disadvantages. They can be quite time-consuming, taking one or two days, and despite their high selectivity and sensitivity, they are designed to be compatible with a particular analyte, which could be a problem in the presence of multiple PAHs, as such a system would only be sensitive to one particular type of them. Some analytes, like Benzo(a)pyrene (BaP), are low in immunogenicity and can occur in a mixture of different PAHs. This fact, added to the small size of these analytes, hinders their detection [72].

Therefore, there is room for improvement by making these techniques detect multiple PAHs in water and soil samples, since, at the moment, they are too specific to single PAH molecules [73].

## 5. Immunosensors

Immunosensors are created through the integration of immunoassay methods in micro-devices. As a compact analytical device, an immunosensor presents a surface where antigen–antibody complex can be detected and also converted into electrical and biomechanical signals using transducers. The final detected signals can then the processed, recorded, and displayed [74]. The advantages of these immunosensors in comparison to traditional PAH detection methods like gas chromatography–mass spectrometry (GC–MS) are several, including miniaturization of instrumental devices. They also need minimal organic compounds, small test samples, little use of organic solvents, and they do not result in extensive cleaning due to potential contamination [75]. Also, the technique makes it possible to analyze many samples at the same time, and in situ analysis of environmental pollutant concentrations in a sample [76,77]. This type of systems would allow a fast screening of multiple samples. The detection of a positive presence of PAHs in the samples would indicate the need for further analysis in the laboratory with more standard analytical techniques.

**Figure 2:**
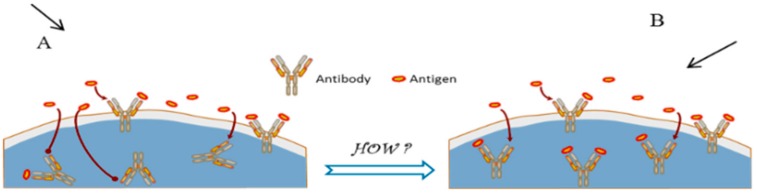
Antibody orientations on the immunosensors. (**A**) Random immobilization; (**B**) oriented immobilization [78].

The standardization of microfabrication techniques in the last 20 years has made the fabrication procedure for immunosensors simple, and the possibility to use surface modification techniques to improve the orientation of the antibodies (Ab) immobilized on them (Figure 2), which makes the detection of PAHs in polluted water sample more effective.

As previously noted, immunosensors are a viable approach to the real-time and fast screening of PAHs [79]. Generally, they are grouped into four groups, depending on the techniques of detection, as shown in Table 4 [80].

**Figure 3:**
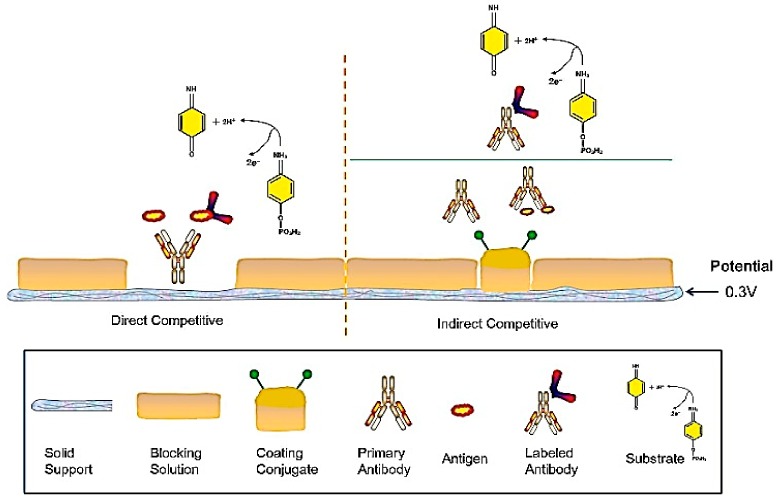
Electrochemical detection using an immunological reaction by measuring the alkaline phosphatase (AP) enzymatic reaction towards the substrate para-aminophenyl phosphate (pAPP). Two electrons were generated with a new product (para-aminophenyl (pAP)) [81].

In this article, we will focus on immunosensors that use electrochemical techniques to quantify the presence and concentration of PAHs, as the diversity of existing sensors is huge. In addition, the use of electrochemical immunosensors presents great advantages in comparison to the above-mentioned methods of detection, as they are easy to fabricate and use, without compromising sensitivity. For example, Ahmad and Moore [81] reported on the performance of an electrochemical immunosensor for the detection of benzo(a)pyrene (BaP) in water samples. Detection was done electrochemically by a common reaction used to create an electrochemical signal in immunological reactions, produced by the substrate para-aminophenyl phosphate (pAPP) (an example of an immunoassay with a pAPP reaction is shown in Figure 3). In this reaction, an alkaline phosphatase enzyme (AP), which is linked to a secondary antibody, is reduced (by hydrolysis of the substrate pAPP) to para-aminophenyl (pAP), freeing hydrogen ions in the process.

The difference in potential created by the two hydrogens is then measured, as it an electrical signal proportional to the presence of the target analyte.

The same authors also used surface functionalization techniques like optimization of surface substrates and active redox probes to increase the sensitivity of the sensor. The surface functionalization techniques mentioned above were demonstrated with the modification of gold surface electrode using 11-MUA SAM solution for the detection of BaP in the water. The electrochemical behavior of the immunosensor was carried out by cyclic voltammetry using potassium ferrocyanide/ferricyanide as a redox probe. The developed immunosensor demonstrated a significant improvement in analytical performance and sensitivity in comparison with the immunosensor with unmodified surface sensors. The performance of 11-MUA SAM in ethanolic solution improved the binding of the protein on the gold surface for the detection of BaP in the water samples. These state-of-the-art techniques of enhancing sensitivity of PAH detection by modifications of the immunosensor will be discussed further.

## 6. Surface Functionalization Techniques

There are several possible surface functionalization techniques for improving PAH detection. However, the current review will focus on three key approaches that are widely discussed in the literature in terms of improving immunosensor performance. These surface functionalization techniques include optimization of substrate surface reaction, redox-active probes, and layer-by-layer technique.

**Figure 4:**
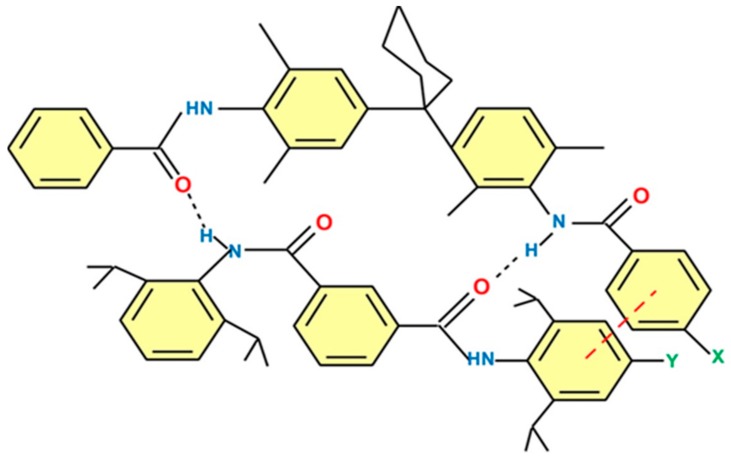
T-shaped π-stacking interactions between (X) and (Y).

First, optimization of surface substrates focuses on improving the sensitivity and selectivity of immunosensors by enhancing antigen–antibody binding and enzymatic reactions [82,83,84]. For example, [83] developed a surface-modified immunosensor using benzo(a)anthracene-7,12-dione/polypyrrole/pyrolytic graphite electrode (BaD/PPy/PGE). The electrochemical behavior of the analyite, benzo(k)fluoranthene (BkF) in the presence of the BaD/PPy/PGE compound was investigated. BkF and BaD have pi stacking (also called π–π interactions; Figure 4), where noncovalent attractive interactions occur between their aromatic rings since they contain pi bonds. As a result of precise *pi*–*pi* interaction between BaD and BkF, the wider linear detection range for BkF achieved was between 1.0 × 10^−12^ and 1.0 × 10^−9^ M with a suitable linearity of R^2^ = 0.9962 and a low limit of detection (1.0 × 10^−13^ M, S/N = 3) were found due to the particular *π*–*π* interactions between BkF and BaD.

**Figure 5:**
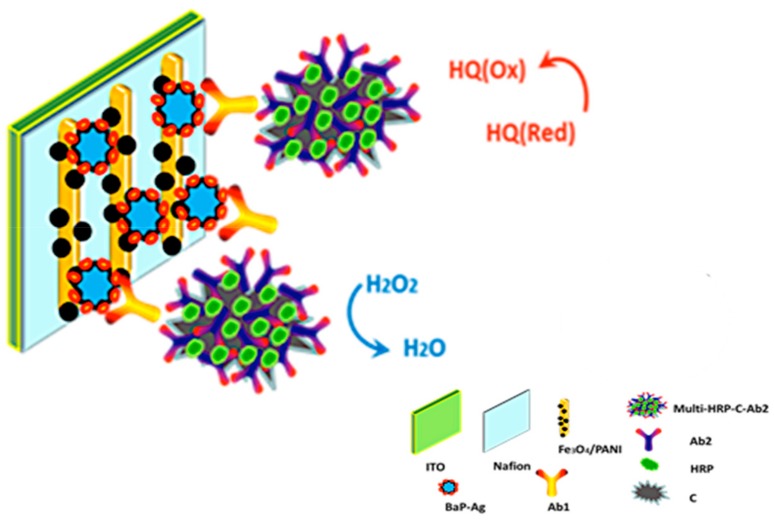
Schematic representation of Fe_3_O_4_/PANI/Nafion-based immunosensor using multi-HRP-HCS-Ab2 bioconjugates as labels [85].

Similarly, the authors of [84] used a biocompatible polyaniline (PANI) layer and iron oxide to develop an electrochemical immunosensor platform. The role of Fe_3_O_4_/PANI on a Nafion/ITO surface is to capture the BaP antigen with the aid of glutaraldehyde. Fabricated multi-HRP-HCS-Ab2 labels were added at the end of the assay (see Figure 5). The technique works through immunoreaction between the BaP antigen and the primary antibody (Ab1) in the sample solution. The authors reported a linear response in the range of 8 pM to 2 nM and a detection limit of 4 pM, which is highly sensitive, compared to conventional PAH detection techniques.

**Figure 6:**
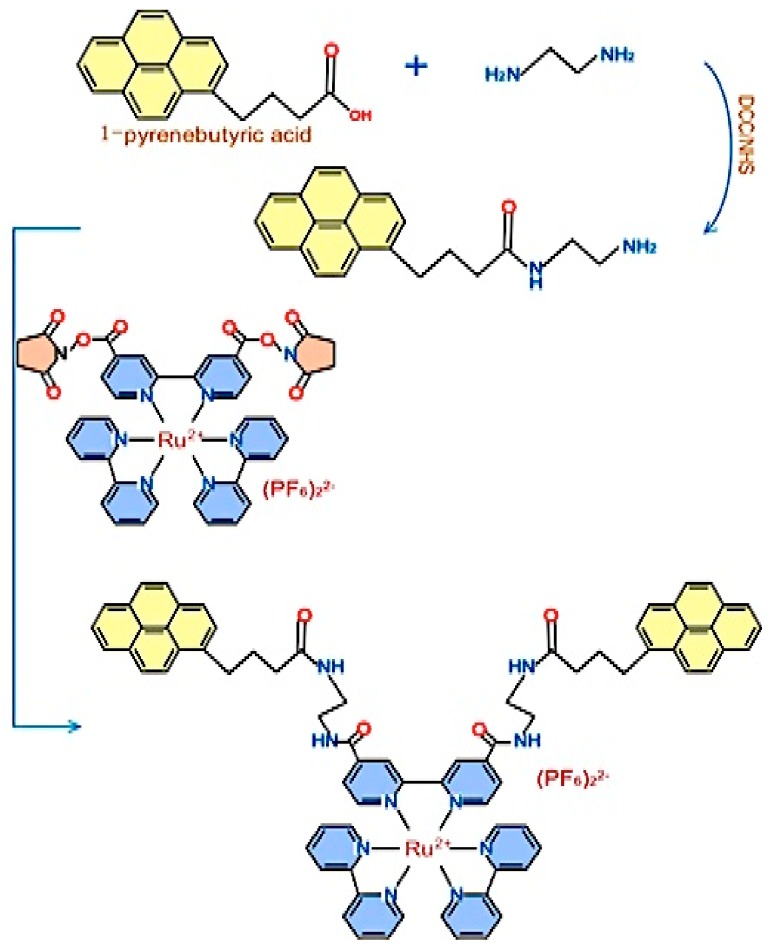
Synthesis of ruthenium tris(bipyridine)-pyrene butyric acid (PAH/Ru) conjugate [87].

A second surface modification technique used in state-of-the-art in this research area is the application of redox-active probes. The electrodes are able to measure the oxidation–reduction potential. The aim of the redox-active probes is to intensify the redox cycling and produce high chemical signals. In immunoassays, small redox molecules are immobilized on the surface of the electrode using antigen/antibody binding, where they are detected directly. The use of this technique has more advantages than the previous highlighted methods of surface enhancement [16], established the redox surface-labeled immunoassay for detecting PAH in water. The model has the potential for detecting low levels of PAHs and Bap to a 2.4 ng mL^−1^ limit. The range of detection decreases with the outcomes of standard immunosensors, whose detection limits ranges between 1.28 g mL^−1^ and 10 ng mL^−1^. Thus, redox probes offer efficient PAHs detection in polluted water, as indicated in Figure 6. In PAH detection, the objective of this technique is to amplify the redox cycling provided by the probes in order to obtain high electrochemical signals [85,86,87].

The common detection range obtained with these probes falls within the results of normal immunosensors, between 1.28 and 10 ng/mL. Therefore, redox probes provide an effective detection of PAHs in polluted water, and the results match the ones gotten from conventional techniques. The use of redox probes is useful as it can simultaneously detect multiple PAHs [87]. Figure 6 shows how a ruthenium tris(bipyridine)-pyrene butyric acid conjugate (PAH/Ru) was synthesized as a redox-labeled tracer [85]. For the PAH/Ru conjugate to be used as a tracer in PAH immunoassays, it needs to be detected by anti-PAH antibodies.

A third alternative technique to surface modification of immunosensors is to develop a layer-by-layer assembly (LbL) [88,89,90]. In LbL, different layers of materials with opposite charges are created to develop an alternative surface using fabrication approaches like electromagnetism, fluidics, immersion, spray, and spin [88]. When LbL assembly is used in nanotubes, it allows the development of multiple bio-interfaces. The sensitivity offered by nanotubes is much higher than materials like dendrimers, metal, and carbon materials [89,90]. These techniques do not come without challenges; LbL deposition may present some disadvantages due to technical issues like obtaining a homogenous coating, irregularly two dimensional (2D) shaped substrates, and difficulties depositing three-dimensional (3D) substrates during LbL assembly [90]. An example of a LbL used in the detection of PAHs in water is a silicon-based device with the platinum-based mercury electrode used as a working electrode and platinum as a supporting electrode integrated into an exterior flow cell arrangement that has a reference electrode [91].

In comparison with other techniques, LbL does not rely on the shape and size of the substrate, hence a wider range of diverse materials can be deposited on various substrates, as shown in the table below. A phospholipid mixture of a triglyceride layer was used to coat mercury and the layer was sensitive to detecting PAHs in seawater samples based on potential difference (see Table 5) [91]. These show how LbL techniques can be applied to improve substrate optimization.

The LbL technique improves sensitivity levels in comparison with other methods such as the optimization of the enzyme substrate [82], since LbL offers a wide linear range from 1.0 to 7 ng/mL, while standard techniques have sensitivity levels of between 1.7 to 9.5 ng/mL. Therefore, it can be concluded that LbL shows equal, if not better sensitivity than conventional PAH detection techniques, and allows multiple detection of PAHs in samples. A common practice of enhancing the LbL immunosensor sensitivity encompasses the combining of proteins with nanomaterials, because the modification of nanomaterial-based surfaces leads to increased sensitivity, wider surfaces, and electrochemical stability [83]. For an illustration of this, recent research efforts have indicated that nanomaterials can be used to detect traces of PAHs in water. An example of an LbL technique used iron oxide nanoparticles and multi-walled carbon nanotubes (MWCNTs), which were incorporated onto calcium alginate beads. MWCNTs ensure there is a large detection surface area, while promoting π interactions with the aromatic rings on PAH samples, obtaining an enhancement of detection limits [92]. Detection of PAHs showed sensitivity in the range of other techniques like optimized surface substrates (limits of detection of 5 ng/L for benzo(a)anthracene and benzo(a)pyrene, and 10 ng/L for benzo(b)fluoranthene, whereas LbL showed typical value 1 × 10^−13^ M.

In comparison with the surface modification techniques, the results of LbL techniques are much better for PAH detection. The sensitivity of LbL in improving the surface of the immunosensor is better than that of substrate enzyme optimization with the detection ranging from 1.0 × 10^−12^ to 1.0 × 10^−9^ M. Thus, the LbL technique provides cheaper and easier procedure for the formation of the multi-layers and facilitates for the integration of different materials within the film structures. LbL can be used to deposit a wide range of material such as biological molecules, ceramics, metals, polyions, and nanoparticles. Also, LbL offers high degree of over thickness control as a result of the variable profile growth of the films that is directly related to the material used, assembly method, and bilayer number [93]. Therefore, this technique can be considered as a versatile nanofabrication method that can be very beneficial in detecting multiple PAHs.

The review of the three surface functionalization techniques explained here indicates that the techniques offer higher detection sensitivity compared to traditional detection methods such as GC/MS. The results for LbL show that its detection limit ranges from 1.0 to 7 ng/mL, while that of the redox-active probes ranges from 1.28 to 10 ng/mL.

This article is a review on the techniques to be able to detect PAHs by immunosensing. It presents different techniques, their level of detection, and the PAHs. A SWOT analysis shows that the process has strengths, weaknesses, opportunities, and threats. First the technique has a higher sensitivity as compared to other processes. However, its range is limited. Despite this threat, it is clear that more improvements can be done for improved results.

## 7. Conclusions

This review provides some of the existing techniques to detect PAH in water environments, more particularly in water sources. The inability of current lab equipment to analyze samples in the field, added to the degree of expertise required to use it and the length of time required for the sample analysis, has led to search for alternative solutions, which we have covered. In addition, the article discussed state-of-the-art surface modification techniques to improve the performance of these sensors. In comparison with the other two techniques, immunosensors based on substrate modification technique show high selectivity, specificity, and sensitivity with wide linear range of PAH detection. Even though the other two techniques may not attain the anticipated high sensitivity, it is important to explore their advantages so that if need be, they can also be implemented. For instance, if they can generate faster results, be implemented easily, or be convenient for analyzing multiple PAHs in a single platform, then they can be adopted in situations that need such conditions. In conclusion, we envision immunosensors as a field-based solution for environmental screening to determine PAHs concentrations in contaminated samples, which could then be further analyzed using GC/MS or HPLC once positive results are detected. The discussed techniques show a high sensitivity that can make them quite competitive in terms of detecting very small traces of PAHs, when compared to traditional techniques such as GC/MS. In addition, they present a versatile platform for rapid and portable analysis of water sources. The advantages, as well as the disadvantage of surface functionalization techniques, and their potential in environmental monitoring were presented.

The findings reveal that of the three techniques, optimized surface substrates may offer higher levels of sensitivity and detection of PAHs, followed by the LbL and redox probes, respectively.

## Figures and Tables

**Table 1:** The effects of polycyclic aromatic hydrocarbons (PAHs) in short and long contacts.

Short	Long-Standing Contact
Impaired lung function and a coronary heart condition by inhalation; intake of water contaminated with PAHs has resulted in diarrhea, vomiting, and nausea conditions; when the human skin is exposed to PAHs, it results in irritation, swelling, and allergic reactions of the skin [11,12,13]	Detrimental effects on the reproductive and development systems; kidney and liver infection; cataracts inducement; jaundice; malfunction of red blood cells (RBCs) [14,15,16]

**Table 2:** The European Union recommended PAHs concentration values in drinking water [22].

Compound	2013/39/EU	
	Annual Average—Environmental Quality Standard Inland Surface Water (µg/L)	Environmental Quality Standard/Required Limit (µg/L)
Anthracene	0.1	0.034
Benzo(a)pyrene	0.00017	0.000057
Benzo(b)fluoranthene	0.00017	0.000057
Benzo(k)fluoranthene	0.00017	0.000057
Benzo(ghi)perylene	0.00017	0.000057
Indeno(123cd)pyrene	0.00017	0.000057
Fluoranthene	0.0063	0.0021
Naphtalene	2	0.67
Pentachlorobenzene	0.007	0.0023

**Table 3:** Published studies of PAHs in various regions and their sources.

PAH Ratio	Value Range	PAHs Source	Country	Ref
IcdP/(IcdP + BghiP)	<0.2	petrogenic	Fraser River basin, Canada	[34]
Flpy/(Flpy + C24Ph)	>0.2–0.5	Pyrolytic	Eagle Harbor, USA	[35]
Ant/(Ant + Phe)	<0.1	petrogenic	Mosel and Saar Rivers in German	[36]
ΣLMW/HMW	>0.75	Pyrolytic	Beijing, China	[37]
Fla/(Fla + Pyr)	<0.1	petrogenic	Yellow River, China.	[38]
Ant/(Ant + Phe) vs. Flur/(Flur + Pyr)	>0.1	Pyrolytic	Egyptian Red Sea coast	[39]
ΣMePhe/Phe	<1	Pyrolytic	Zaragoza city, Spain	[40]
Ind/Ind + B(g,h,i)P	>1	petrogenic	Khao Lak coastal area, Thailand	[41]

Table of Acronyms: There are various; Ant/(Ant + Phe)—anthracene/(anthracene, plus phenanthrene); Fla/(Fla + Pyr)—fluoranthene/(fluoranthene plus pyrene); Flpy/C24ph—fluoranthene plus pyrene/C2-4 phenanthrene; Flpy/(Flpy + C24ph)—fluoranthene/(fluoranthene,plus C2-4 phenanthrenes; Flur/(Flur + Pyr)—fluoranthene/(fluoranthene plus pyrene); IcdP/(IcdP + BghiP)—indeno(1,2,3-cd)pyrene/indeno(1,2,3-cd)pyrene plus benzo(g,h,i)perylene; Ind/Ind + B(g,h,i)P—indeno/(indeno plus benzo(g,h,i)perylene); ΣMePhe/Phe—sum of methyl-phenanthrene/phenanthrene; and ΣLMW/HMW—sum of low molecular weight/sum of high molecular weight.

**Table 4:** Groups of immunosensors and how they are defined [78].

Immunosensors	Definitions
Electrochemical	An antibody can be used as a receptor and can be grouped depending on the detection method [66,79], such as electrochemical impedance spectroscopy, potentiometric, conductometric, or amperometric [80,81,82,83].
Optical	The antibody–antigen complex triggers changes in the optical characteristics of the substrate, which can be detected by the use of different techniques, such as total internal reflection fluorescence (TIRF) and polarization–modulation infrared reflection–absorption spectroscopy (PM-IRRAS) [76]. Additional methods of detection include chemiluminescence, fluorescence [77], and Raman spectroscopy [73].
Mechanical	The basis in of this type of transduction is the response of a surface upon variations in the stress and loading applied to it. Velocity and position can be used in detecting measurement performance [74,84]. In addition, piezoelectric materials (where mechanical stress generates an accumulation of electric charge) such as quartz crystals have been used to immobilize antibodies and antigens [76,81].

**Table 5:** Detection limits of phenanthrene, pyrene, anthracene, and fluoranthene [91].

PAHs	Detection Limit (μg/L)
Phenanthrene	0.33
Pyrene	0.35
Anthracene	0.15
Fluoranthene	0.32

## References

[1] Baklanov A., Hänninen O., Slørdal L.H., Kukkonen J., Bjergene N., Fay B. (2007). Integrated systems for forecasting urban meteorology, air pollution and population exposure. Atmos. Chem. Phys..

[2] Latimer J., Zheng J., Douben P.E.T. (2003). The Sources, Transport, and Fate of PAH in the Marine Environment. PAHs: An Ecotoxicological Perspective.

[3] Menzie A.C., Potocki B.B., Santodonato J. (1992). Exposure to carcinogenic PAHs in the environment. Environ. Sci. Technol..

[4] Arey J., Atkinson R., Douben P.E.T. (2003). Photochemical reactions of PAH in the atmosphere. PAHs: An Ecotoxicological Perspective.

[5] Di Toro D.M., McGrath J.A., Hansen D.J. (2000). Technical basis for narcotic chemicals and polycyclic aromatic hydrocarbon criteria. I. Water and tissue. Environ. Toxicol. Chem..

[6] Kim K., Jahan S.A., Kabir E., Brown C.J. (2013). A review of airborne polycyclic aromatic hydrocarbons (PAHs) and their human health effects. Environ. Int..

[7] Armstrong B.G., Hutchinson E., Unwin J., Fletcher T. (2004). Lung cancer risk after exposure to polycyclic aromatic hydrocarbons: A review and meta-analysis. Environ. Health Perspect..

[8] Canadian Council of Ministers of the Environment (2010). Canadian Soil Quality Guidelines for Potentially Carcinogenic and Other PAHs: Scientific Criteria Document.

[9] Suess M.J. (1976). The environmental load and cycle of polycyclic aromatic hydrocarbons. Sci. Total Environ..

[10] European Communities (2016). The quality of water intended for human consumption. Off. J. Eur. Communities.

[11] Polycyclic aromatic hydrocarbons (PAHs) biologic exposure indices (BEI). https://www.acgih.org/docs/default-source/presentations/2005/aihce2005_presentation.pdf?sfvrsn=6afadf0d_2.

[12] Unwin J., Cocker J., Scobbie E.H. (2006). An assessment of occupational exposure to polycyclic aromatic hydrocarbons in the UK. Ann. Occup. Hyg..

[13] International Programme on Chemical Safety (2010). Polycyclic Aromatic Hydrocarbons, Selected Non-Heterocyclic. http://www.inchem.org/documents/ehc/ehc/ehc202htm.

[14] Bach B.P., Kelley J.M., Tate C.R., McCrory C.D. (2003). Screening for lung cancer: A review of the current literature. Chest.

[15] Diggs D.L., Huderson A.C., Harris K.L., Myers J.N., Banks L.D., Rekhadevi P.V. (2011). Polycyclic aromatic hydrocarbons and digestive tract cancers: A perspective. J. Environ. Sci. Health. Part C.

[16] Olsson A.C., Fevotte J., Fletcher T., Cassidy A., Mannetje A., Brennan P. (2010). Occupational exposure to polycyclic aromatic hydrocarbons and lung cancer risk: A multicenter study in Europe. Occup. Environ. Med..

[17] Jafarabadi A.R., Bakhtiari A.R., Yaghoobi Z., Yap C.K., Maisano M., Cappello T. (2019). Distributions and compositional patterns of polycyclic aromatic hydrocarbons (PAHs) and their derivatives in three edible fishes from Kharg coral Island, Persian Gulf, Iran. Chemosphere.

[18] US Environmental Protection Agency (2008). Polycyclic Aromatic Hydrocarbons (PAHs)—EPA Fact Sheet.

[19] Burchiel S.W., Luster M.I. (2001). Signaling by environmental polycyclic aromatic hydrocarbons in human lymphocytes. Clin. Immunol..

[20] Andersson J.T., Achten C. (2015). Time to say goodbye to the 16 EPA PAHs? Toward an up-to-date use of PACs for environmental purposes. Christine Polycycl. Aromat. Compd..

[21] Rajasärkkä J., Pernica M., Kuta J., Lašňák J., Šimek Z., Bláha L. (2016). Drinking water contaminants from epoxy resin-coated pipes: A field study. Water Res..

[22] The European Water Framework Directive (2013). Introduction to EU WFD 2013/39/EU. Thermo Scientific Environmental Solutions Reference Guide.

[23] Nowacka A., Włodarczyk-Makuła M. (2013). Monitoring of polycyclic aromatic hydrocarbons in water during preparation processes. Polycycl. Aromat. Compd..

[24] Maliszewska-Kordybach B. (1999). Sources, concentrations, fate and effects of polycyclic aromatic hydrocarbons (PAHs) in the environment. Part A: PAHs in air. Pol. J. Environ. Stud..

[25] Akyuz M., Cabuk H. (2010). Gas—particle partitioning and seasonal variation of polycyclic aromatic hydrocarbons in the atmosphere of Zonguldak, Turkey. Sci. Total Environ..

[26] Determination of Parent and Alkylated Polycyclic Aromatic Hydrocarbons (PAHs) in Biota and Sediment. https://pdfs.semanticscholar.org/c83a/9554342d38c4eeab6004b44b9b285278714d.pdf.

[27] Masih J., Singhvi R., Kumar K., Jain V.K. (2012). Seasonal variation and sources of polycyclic aromatic hydrocarbons (PAHs) in indoor and outdoor air in a semi-arid tract of northern India. Aerosol Air Qual Res..

[28] Wild S.R., Jones K.C. (1995). Polynuclear aromatic hydrocarbons in the United Kingdom environment: A preliminary source inventory and budget. Environ. Pollut..

[29] Sarria-Villa R., Ocampo-Duque W., Páez M., Schuhmacher M. (2016). Presence of PAHs in water and sediments of the Colombian Cauca River during heavy rain episodes, and implications for risk assessment. Sci. Total Environ..

[30] Singare P.U. (2015). Studies on polycyclic aromatic hydrocarbons in surface sediments of Mithi River near Mumbai, India: Assessment of sources, toxicity risk and biological impact. Mar. Pollut. Bull..

[31] Pérez-Fernández B., Viñas L., Franco M.Á., Bargiela J. (2015). PAHs in the Ría de Arousa (NW Spain): A consideration of PAHs sources and abundance. Mar. Pollut. Bull..

[32] Liu G., Song D., Chen F. (2013). Towards the fabrication of a label-free amperometric immunosensor using SWNTs for direct detection of paraoxon. Talanta.

[33] Zhang Z., Huang J., Yu G., Hong H. (2004). Occurrence of PAHs, PCBs and organochlorine pesticides in the Tonghui River of Beijing, China. Environ. Pollut..

[34] Yunker M.B., Macdonald R.W., Vingarzan R., Mitchell R.H., Goyette D., Sylvestre S. (2000). PAHs in the Fraser River basin: A critical appraisal of PAH ratios as indicators of PAH source and composition. Org. Geochem..

[35] Neff J., Stout S., Gunster D. (2004). Ecological Risk Assessment of Polycyclic Aromatic Hydrocarbons in Sediments: Identifying Sources and Ecological Hazard. Integr. Environ. Assess. Manag..

[36] Pies C., Hoffmann B., Petrowsky J., Yang Y., Ternes T.A., Hofmann T. (2008). Characterization and source identification of polycyclic aromatic hydrocarbons (PAHs) in river bank soils. Chemosphere.

[37] Zhang W., Zhang S., Wan C., Yue D., Ye Y., Wang X. (2008). Source diagnostics of polycyclic aromatic hydrocarbons in urban road runoff, dust, rain and canopy throughfall. Environ. Pollut..

[38] Ding S., Xu Y., Wang Y., Zhang X., Zhao L., Ruan J., Wu W. (2014). Spatial and Temporal Variability of Polycyclic Aromatic Hydrocarbons in Sediments from Yellow River-Dominated Margin. Sci. World J..

[39] El Nemr A., Moneer A., Ragab S., El Sikaily A. (2016). Distribution and sources of n-alkanes and polycyclic aromatic hydrocarbons in shellfish of the Egyptian Red Sea coast. Egypt. J. Aquat. Res..

[40] Callén M., de la Cruz M., López J., Mastral A. (2011). PAH in airborne particulate matter: Carcinogenic character of PM10 samples and assessment of the energy generation impact. Fuel Process. Technol..

[41] Tipmanee D., Deelaman W., Pongpiachan S., Schwarzer K., Sompongchaiyakul P. (2012). Using Polycyclic Aromatic Hydrocarbons (PAHs) as a chemical proxy to indicate Tsunami 2004 backwash in Khao Lak coastal area, Thailand. Nat. Hazards Earth Syst. Sci..

[42] Jiang Y., Zhang X., Zhang Z. (2018). Co-biodegradation of pyrene and other PAHs by the bacterium Acinetobacter johnsonii. Ecotoxicol. Environ. Saf..

[43] Dong C.D., Chen C.F., Chen C.W. (2012). Determination of polycyclic aromatic hydrocarbons in industrial harbor sediments by GC-MS. Int. J. Environ. Res. Public Health.

[44] Atkinson R. (1987). A structure-activity relationship for the estimation of rate constants for the gas-phase reactions of OH radicals with organic compounds. Int. J. Chem. Kinet..

[45] Elcoroaristizabal S., de Juan A., García J., Durana N., Alonso C.L. (2014). Comparison of second-order multivariate methods for screening and determination of PAHs by total fluorescence spectroscopy. Chem. Intel. Lab. Syst..

[46] Molaei S., Saleh A., Ghoulipour VSeidi S. (2016). Centrifuge-less Emulsification Microextraction Using Effervescent CO_2_ Tablet for On-site Extraction of PAHs in Water Samples Prior to GC–MS Detection. Chromatographia.

[47] Wang W., Ma R., Wu Q., Wang C., Wang Z. (2013). Magnetic microsphere-confined graphene for the extraction of polycyclic aromatic hydrocarbons from environmental water samples coupled with high performance liquid chromatography–fluorescence analysis. J. Chromatogr. A.

[48] Gilgenast E., Boczkaj G., Przyjazny A., Kamiński A. (2011). Sample preparation procedure for the determination of polycyclic aromatic hydrocarbons in petroleum vacuum residue and bitumen. Anal. Bioanal. Chem..

[49] Lux G., Langer A., Pschenitza M., Karsunke X., Strasser R., Niessner R., Knopp D., Rant U. (2015). Detection of the carcinogenic water pollutant benzo [a] pyrene with an electro-switchable biosurface. Anal. Chem..

[50] Zhang W., Liu Q.X., Guo Z.H., Lin J.H. (2018). Practical Application of Aptamer-Based Biosensors in Detection of Low Molecular Weight Pollutants in Water Sources. Molecules.

[51] Manoli E., Kouras A., Samara C. (2004). Profile analysis of ambient and source emitted particle-bound polycyclic aromatic hydrocarbons from three sites in northern Greece. Chemosphere.

[52] Mostert M., Ayoko G.A., Kokot S. (2010). Application of chemometrics to analysis of soil pollutants. Trends Anal. Chem..

[53] Hwang H., Wade T., Sericano J. (2003). Concentrations and source characterization of polycyclic aromatic hydrocarbons in pine needles from Korea, Mexico, and United States. Atmos. Environ..

[54] Kielhorn J., Boehncke A. (1998). Polynuclear Aromatic Hydrocarbons. Guidelines for Drinking-Water Quality.

[55] Vo-Dinh T., Fetzer J., Campiglia A. (1998). Monitoring and characterization of polyaromatic compounds in the environment. Talanta.

[56] Hund K., Traunspurger W. (1994). Ecotox-evaluation strategy for soil bioremediation exemplified for a PAH-contaminated site. Chemosphere.

[57] Hamza-Chaffai A. (2014). Usefulness of bioindicators and biomarkers in pollution biomonitoring. Int. J. Biotechnol. Wellness Ind..

[58] Del Carlo M., Di Marcello M., Perugini M., Ponzielli V., Sergi M., Mascini M., Compagnone D. (2008). Electrochemical DNA biosensor for polycyclic aromatic hydrocarbon detection. Microchim. Acta.

[59] Gu M.B., Chang S.T. (2001). Soil biosensor for the detection of PAH toxicity using an immobilized recombinant bacterium and a biosurfactant. Biosens. Bioelectron..

[60] Song Y., Jiang B., Tian S., Tang H., Liu Z., Li C., Li G. (2014). A whole-cell bioreporter approach for the genotoxicity assessment of bioavailability of toxic compounds in contaminated soil in China. Environ. Pollut..

[61] Jones J., Anderson J.W., Tukey R.H. (2000). Using the metabolism of PAHs in a human cell line to characterize environmental samples. Environ. Toxicol. Pharm..

[62] Fent K. (2001). Fish cell lines as versatile tools in ecotoxicology: Assessment of cytotoxicity, cytochrome P4501A induction potential and estrogenic activity of chemicals and environmental samples. Toxicol. Vitr..

[63] Ahmad A., Moore E. (2009). Comparison of Cell-Based Biosensors with Traditional Analytical Techniques for Cytotoxicity Monitoring and Screening of Polycyclic Aromatic Hydrocarbons in the Environment. Anal. Lett..

[64] Anderson J.W., Zeng E., Jones J.M. (1999). Correlation between the response of a human cell line (P450RGS) and the distribution of sediment PAHs and PCBs on the Palos Verdes Shelf, California. Environ. Toxicol. Chem..

[65] Lawal A.T. (2017). Polycyclic aromatic hydrocarbons. A review. Cogent Environ. Sci..

[66] Srogi K. (2007). Monitoring of environmental exposure to polycyclic aromatic hydrocarbons: A review. Environ. Chem. Lett..

[67] Engvall E., Jonsson K., Perlmann P. (1971). Enzyme-linked immunosorbent assay. II. Quantitative assay of protein antigen, immunoglobulin G, by means of enzyme-labelled antigen and antibody-coated tubes. Biochim. Biophys. Acta.

[68] Bansal V., Kumar P., Kwon E.E., Kim K.H. (2017). Review of the quantification techniques for polycyclic aromatic hydrocarbons (PAHs) in food products. Crit. Rev. Food Sci. Nutr..

[69] Fillmann G., Bicego M.C., Zamboni A., Fileman T.W., Depledge M.H., Readman J.W. (2016). Readma Validation of immunoassay methods to determine hydrocarbon contamination a in estuarine Sediments. J. Braz. Chem. Soc..

[70] National Environmental Methods Index (NEMI). https://acwi.gov/methods/pubs/nemi_pubs/nemi_fs2.pdf.

[71] National Environmental Methods Index (NEMI) (2018). Rapid Assay PAH Test Kit A00156/A00157.

[72] National Environmental Methods Index (NEMI) (2018). EPA Method 70620.

[73] Hennion M.C., Barcelo D. (1998). Strengths and limitations of immunoassays for effective and efficient use for pesticide analysis in water samples: A review. Anal. Chim. Acta.

[74] Pollap A., Kochana J. (2019). Electrochemical Immunosensors for Antibiotic Detection. Biosensor.

[75] Rhouati A., Catanante G., Nunes G., Hayat A., Marty J.L. (2016). Label-Free Aptasensors for the Detection of Mycotoxins. Sensors.

[76] Fähnrich K., Pravda M., Guilbault G. (2002). Immunochemical detection of polycyclic aromatic hydrocarbons (PAHs). Anal. Lett..

[77] Ramírez N.B., Salgado A.M., Valdman B. (2009). The evolution and developments of immunosensors for health and environmental monitoring: Problems and perspectives. Braz. J. Chem. Eng..

[78] Trilling A.K., Beekwilder J., Zuilhof H. (2013). Antibody orientation on biosensor surfaces: A mini review. Analyst.

[79] Zhang Y.H., Su Q., Xu J.H., Zhang Y., Chen S.T. (2014). Detecting of Benzo [a] pyrene Using a Label-free Amperometric Immunosensor. Int. J. Electrochem. Sci..

[80] Sassolas A., Prieto-Simón B., Marty J.L. (2012). Biosensors for pesticide detection: New trends. Am. J. Anal. Chem..

[81] Ahmad A., Moore E. (2012). Electrochemical immunosensor modified with self-assembled monolayer of 11-mercaptoundecanoic acid on gold electrodes for detection of benzo [a] pyrene in water. Analyst.

[82] Zheng X., Tian D., Duan S., Wei M., Liu S., Zhou C., Wu G. (2014). Polypyrrole composite film for highly sensitive and selective electrochemical determination sensors. Electrochim. Acta.

[83] Nanomaterials for advancing the health immunosensor. https://www.intechopen.com/books/biosensors-micro-and-nanoscale-applications/nanomaterials-for-advancing-the-health-immunosensor.

[84] Barathi P., Senthil Kumar A. (2013). Electrochemical conversion of unreactive pyrene to highly redox-active 1, 2-quinone derivatives on a carbon nanotube-modified gold electrode surface and its selective hydrogen peroxide sensing. Langmuir.

[85] Lin M., Liu Y., Sun Z., Zhang S., Yang Z., Ni C. (2012). Electrochemical immunoassay of benzo [a] pyrene based on dual amplification strategy of electron-accelerated Fe_3_O_4_/polyaniline platform and multi-enzyme-functionalized carbon sphere label. Anal. Chim. Acta.

[86] Akanda M.R., Aziz M.A., Jo K., Tamilavan V., Hyun M.H., Kim S., Yang H. (2011). Optimization of phosphatase-and redox cycling-based immunosensors and its application to ultrasensitive detection of troponin I. Anal. Chem..

[87] Wei M.Y., Wen S.D., Yang X.Q., Guo L.H. (2009). Development of redox-labeled electrochemical immunoassay for polycyclic aromatic hydrocarbons with controlled surface modification and catalytic voltammetric detection. Biosens. Bioelectron..

[88] Zhang Z., Cong Y., Huang Y., Du X. (2019). Nanomaterials-Based Electrochemical Immunosensors. Micromachines.

[89] Yost A.L., Shahsavari S., Bradwell G.M., Polak R., Fachin F., Cohen R.E., Wardle B.L. (2015). Layer-by-layer functionalized nanotube arrays: A versatile microfluidic platform for biodetection. Microsyst. Nanoeng..

[90] Lin H.L., Li Z.H., Liu P., Song B.B., Cai Q.Y., Grimes C.A. (2016). Aminocalix [4] arene monolayers as magnetoelastic sensor sensing elements for selective detection of benzo [a] pyrene. Anal. Methods.

[91] Penezić A., Gašparović B., Stipaničev D., Nelson A. (2014). In situ electrochemical method for detecting freely dissolved polycyclic aromatic hydrocarbons in water. Environ. Chem..

[92] Bunkoed O., Kanatharana P. (2015). Extraction of polycyclic aromatic hydrocarbons with a magnetic sorbent composed of alginate, magnetite nanoparticles and multiwalled carbon nanotubes. Microchim. Acta.

[93] Mehdinia A., Khodaee N., Jabbari A. (2015). Fabrication of graphene/Fe_3_O_4_@ polythiophene nanocomposite and its application in the magnetic solid-phase extraction of polycyclic aromatic hydrocarbons from environmental water samples. Anal. Chim. Acta.

